# A Systematic Review on the Phytoremediation Potential of Grass Species in Ethiopia

**DOI:** 10.1155/tswj/9434923

**Published:** 2026-06-13

**Authors:** Endalamaw Yihune, Amare Bitew Mekonnen, Getahun Yemata

**Affiliations:** ^1^ Department of Biology, College of Science, Bahir Dar University, P.O. Box 79, Bahir Dar, Ethiopia, bdu.edu.et

**Keywords:** Ethiopia, grasses, heavy metals, phytoextraction, phytoremediation

## Abstract

Environmental contamination has shown an increasing trend due to urbanization, industrialization, and the intensive use of pesticides and fertilizers in Ethiopia. In response to this, phytoremediation has gained attention as an inexpensive and eco‐friendly approach to the protection of soil, water, and air from contamination. However, scientific works that reviewed studies in the area are scarce. Thus, this review is aimed at compiling findings and evaluating the potential role of grass in phytoremediation in Ethiopia. Information of this type was retrieved from international scientific databases of Web of Science, PubMed, Scopus, and Google Scholar. Keywords include “native grasses,” “phytoremediation,” “Ethiopia,” and “heavy metals.” The data was extracted on the grass species, the phytoremediation mechanism, the bioaccumulation factor, the translocation factor, biomass production, growth rate, and others. This systematic review attempts to gather articles on grasses used for phytoremediation in Ethiopia. Following PRISMA guidelines, relevant keywords were used to search articles. A total of 17 grass species have been recognized for their phytoremediation ability. The results found that grass species such as *Chrysopogon zizanioides*, *Rumex nepalensis*, *Chloris gayana*, and *Cyperus* spp. are highly used for phytoremediation. This review confirms that Ethiopia′s indigenous grassland is a precious and still relatively underexploited resource for developing cost‐effective environmental improvement practices. Thus, it is highly recommended that future efforts focus on large‐scale in situ field trials using grass species and explore methods to maximize remediation effectiveness.

## 1. Introduction

Heavy metals, pesticides, and industrial waste have increased rapidly due to various natural processes and anthropogenic activities. They have severe detrimental effects on the ecosystem and human health and require sustainable remediation strategies such as phytoremediation [1]. Conventional remediation methods, such as excavation and chemical treatment, are often costly and disrupt the environmental equilibrium. Phytoremediation is the use of plants to remove, render, or degrade environmental pollutants and has gained international attention as an inexpensive and environmentally friendly approach to soil, water, and air contamination [2]. Phytoremediation has emerged as an effective and sustainable approach for environmental remediation due to its relatively low cost and minimal ecological disturbance [3]. Numerous grass species have been investigated for their phytoremediation potential worldwide, including hyperaccumulators such as *Brassica juncea* and *Helianthus annuus*, which are capable of extracting and immobilizing various environmental contaminants [4]. However, the efficiency of phytoremediation varies with the adaptability of the plant, the biomass yield, and the tolerance to pollutants, which again depends on the species and surrounding conditions [5]. In recent years, there has been an expansion in research in the study of native plant species due to ecological compatibility and resilience within their own environment [6].

In Ethiopia, environmental pollution with heavy metals from industrialization, mining, and agricultural intensification is now a concern [7]. In some urban and semiurban areas, heavy metal pollution from textile industries, tanneries, and untreated wastewater discharges has been confirmed [8]. Furthermore, the inappropriate use of agricultural inputs such as pesticides and fertilizers, including floriculture, has aggravated soil, water, and air pollution, thus threatening biodiversity and food security [9]. Despite these concerns, studies and reviews of finding sustainable ways of managing environmental pollution, such as phytoremediation, are limited. There have been insignificant studies on the role of diverse Ethiopian flora, particularly indigenous grass species, in phytoremediation in the areas of soil, household running water, and air treatment in different environments. Native grasses are well adapted to local climatic and ecological conditions and often demonstrate higher survival and restoration efficiency than exotic species in ecological rehabilitation programs [10, 11]. In many developing countries, including Ethiopia, land degradation has become a major environmental concern due to soil erosion, unsustainable land use practices, and increasing anthropogenic pressures, which significantly reduce soil fertility and ecosystem productivity [12]. As a result, the use of indigenous plant species for phytoremediation and ecological restoration has gained increasing attention. Several native and widely distributed grass species have shown promising potential for the remediation of contaminated environments. For instance, *Cynodon dactylon* has been reported to accumulate heavy metals such as lead (Pb) and cadmium (Cd) from contaminated soils [13]. Similarly, elephant grass (*Pennisetum purpureum*) has been evaluated for its capacity to stabilize chromium (Cr) in contaminated soils. Therefore, this review is aimed at providing a comprehensive overview of the current knowledge on grass species with phytoremediation potential by synthesizing information on studied species, target pollutants (heavy metals and organic contaminants), and their underlying remediation mechanisms. Furthermore, the review identifies existing research gaps and proposes future research directions, including the exploration of additional grass species, strategies to enhance remediation efficiency, and the development of practical implementation guidelines for the sustainable use of grasses in the remediation of contaminated soil, water, and air in Ethiopia.

## 2. Methodology

A comprehensive literature search was conducted in Web of Science, Scopus, PubMed, and Google Scholar to identify studies related to the phytoremediation potential of grass species in Ethiopia published between 2000 and 2024. In Web of Science, the following topic search (TS) string was used: TS = “phytoremediation” OR “phytoextraction” OR “phytostabilization” OR “phytodegradation” AND “grass species” OR “grasses” OR “Poaceae” AND “Ethiopia” OR “Ethiopian.” In Scopus, the search was performed using title (“phytoremediation” OR “phytoextraction” OR “phytostabilization” OR “phytodegradation” AND (“grass species” OR “grasses” OR “Poaceae”) AND (“Ethiopia” OR “Ethiopian”). In PubMed, the search string applied was “phytoremediation” OR “phytoextraction” OR “phytostabilization” OR “phytodegradation” AND “grass species” OR “grasses” OR “Poaceae” AND “Ethiopia” OR “Ethiopian.” In Google Scholar, the following keyword combination was used: “phytoremediation” OR “phytoextraction” OR “phytostabilization” AND “grass species” OR “grasses” OR “Poaceae” AND “Ethiopia” OR “Ethiopian.” Boolean operators were used to combine keywords related to phytoremediation and grass species. The search was performed using combinations of keywords such as “phytoremediation,” “native grasses,” “Ethiopia,” and specific pollutant names including heavy metals, Cr, Pb, and Cd [14, 15].

Articles were selected based on predefined inclusion and exclusion criteria. Studies were included if they focused on phytoremediation using grass species, particularly native and widely distributed grasses relevant to Ethiopia, and reported experimental as well as review‐based evidence on pollutant removal or stabilization. Studies not related to phytoremediation, nongrass plant species, or lacking sufficient methodological and experimental information were excluded. Only peer‐reviewed articles written in English and published in reputable scientific journals were considered for the review. Furthermore, data from bioconcentration factor (BCF), translocation factor (TF), biomass production, growth rate, phytoextraction, and phytostabilization data were obtained [16]. The number of records identified, screened, excluded, and ultimately included in the review process was determined using the PRISMA flow diagram.

### 2.1. Data Extraction and Identification

**Figure 1:**
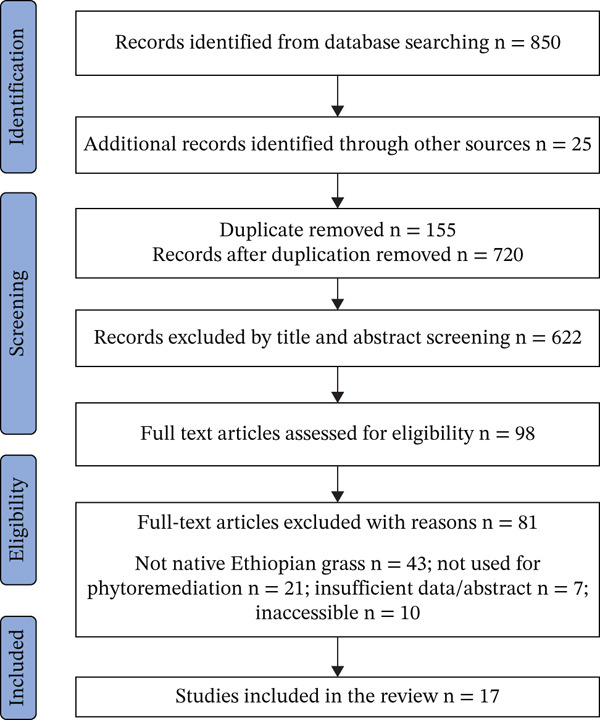
PRISMA diagram: phytoremediation potential of grass species in Ethiopia.

The complete review process began with a robust search strategy across multiple databases using comprehensive keywords, which yielded a large number of records. These records were then accurately screened according to predefined inclusion and exclusion criteria, filtering out irrelevant studies (Figure 1). The remaining articles were assessed in full text for eligibility, where those that meet the inclusion criteria, such as focusing on the phytoremediation potential of grass species in Ethiopia, are selected for data extraction. This process used a structured method to extract key details such as study information, grass species, phytoremediation potential of grass species, and methodological data, ensuring consistency and accuracy. Finally, the extracted data were organized, standardized, and synthesized to interpret trends, generate a narrative summary, present results in tables and figures, and address research questions, ending with conclusions and recommendations for future research.

## 3. Result and Discussion

### 3.1. Grass Species Used for Phytoremediation

The study assessed the phytoremediation potential of various native grass species in Ethiopia. In the present study, a total of 17 grass species were identified and found to have various roles in the control of environmental pollution from different sources (Table 1). In this regard, *Vetiveria zizanioides* was found to be effective in wastewater treatment with significant phytoextraction and phytostabilization potential. This grass species showed high tolerance, removal of N and P from wastewater, and a bioaccumulation factor greater than one. Similarly, Jiru and Wari [24], Teklu et al. [28], and Yohannes et al. [18] have studied the potential of Vetiver grass (*Vetiveria zizanioides*) in wastewater treatment. The findings indicate its effectiveness in removing a broad spectrum of heavy metals, including zinc, copper, nickel, Cr, Pb, and Cd, from contaminated soil, air, and water. The study found that Vetiver grass has shown the most promising phytoremediation role in constructed wetlands for the treatment of wastewater from coffee processing plants, demonstrating high removal rates for nutrients such as nitrogen and phosphorus [18]. Furthermore, these studies suggested its potential for phytostabilizing Cr (VI) (indicated by a bioaccumulation factor greater than one) and phytoextracting manganese and nickel (indicated by a TF greater than one) [20, 21].

**Table 1:** Phytoremediation potential of grass species in Ethiopia.

Scientific name	Common name	Targeted pollutants	Phytoremediation mechanism	Key findings	References
*Chrysopogon zizanioides* (L.) Roberty	Vetiver grass	Zn, Cu, Ni, Cr, Pb, and Cd	Phytoextraction and phytostabilization	High tolerance, effective in wastewater treatment (high removal of N and P), phytostabilization of Cr (VI) (BAF > 1 mg kg^−1^), and Mn and Ni phytoextraction (TF > 1 mg kg^−1^)	Gebru, [17], Yohannes et al. [18]
*Chloris gayana* Kunth	Rhode grass	Zn, Cu, Mn, and micronutrients	Phytoextraction	Uptake of Zn, Cu, and Mn from leachate, high biomass in marginal soils, and higher micronutrient accumulation than in oat	Seyoum & Dawit [19]
*Setaria sphacelata*	Forage setaria	Cu, Fe, Mn, and Zn	Phytoextraction	Higher micronutrient accumulation than in oat and more effective in removing quantity per unit area	Seyoum, & Dawit [19]
*Arundo donax* L.	Giant reed	Cr (VI), Cd, Cr, and Pb	Phytoextraction	Phytoextraction of Cr (VI) (TF > 1 mg kg^−1^) and Cd, Cr, and Pb absorption from river water	Gebretekle et al. *[* *20* *]*, Gebru [21]
*Argemone mexicana* L.	Argemone mexicana	Cu and Ni	Phytoextraction	Potential for Cu and Ni removal around the tailings dam	Tolessa et al. *[* *16* *]*
*Rumex nepalensis* Spreng.	Nepali dock	Cu, Ni, and Zn	Phytoextraction (Cu and Ni) and phytostabilization (Zn)	Accumulates Ni in aboveground parts, potential for Cu, Ni phytoextraction, and Zn phytostabilization	Tolessa et al. [16]
*Cyperus alopecuroides* Rottb.	Foxtail flatsedge	Cu, Ni, and Zn	Phytoextraction (Cu and Ni) and phytostabilization (Zn)	Accumulates Ni in aboveground parts, potential for Cu, Ni phytoextraction, and Zn phytostabilization	Tolessa et al. [16]
*Schoenoplectiella confusa* (N.E.Br.) J.R.Starr	*Schoenoplectus confusus*	Cu	Phytoextraction	Potential for Cu removal around the tailings dam	Tolessa et al. [16]
*Typha latifolia* L.	Elephant grass	Zn	Phytostabilization	Ability for Zn phytostabilization around the tailings dam	Tolessa et al. *[* *16* *]*
*Panicum maximum* L.	Guinea grass	Pb, Cr, and Cd	Phytoextraction	Promising for the remediation of contaminated soils	Olatunji et al. *[* *22* *]*
*Pennisetum pedicellatum*	Desho grass		Phytostabilization	Effective for soil bund stabilization and erosion control	Mekuria et al. *[* *23* *]*
*Cenchrus purpureus* (Schumach.) Morrone	Elephant grass	Cu	Phytoextraction	Copper uptake from leachate and soil bund stabilization	Jiru and Wari [24]
*Brachiaria humidicola*	Chomo grass		Phytostabilization	Role in degraded land rehabilitation and soil fertility improvement	Hundera and Tadesse [25]
*Setaria incrassata*	Purple pigeon grass		Phytostabilization	Used for erosion control	Seyoum & Dawit [19]
*Lemna minor*	Duckweed	Heavy metals (Co, Cd, Zn, Ni, Cu, Fe, Mn, and Cr), COD, BOD, TDS, phosphorus, and nitrogen	Phytoextraction and rhizofiltration	High accumulator for Fe, Mn, Zn, and Co, high BCF for Mn and Fe, and effective in removing organic matter and nutrients from wastewater	Amare et al. *[* *26* *]*
*Azolla filiculoides*	Water fern	Heavy metals (Co, Cd, Zn, Ni, Cu, Fe, Mn, and Cr), COD, BOD, TDS, phosphorus, and nitrogen	Phytoextraction and rhizofiltration	High accumulator for Fe, Mn, Zn, and Cu, high BCF for Mn and Fe, and effective in removing organic matter and nutrients from wastewater	Amare et al. *[* *26* *]*
*Cyperus papyrus*	Dengel	COD, BOD, and (NH4+)	Phytoextraction and rhizofiltration	Effective in removing organic matter and nutrients from wastewater	Hamad [27]

Similarly, *Chloris gayana* has also demonstrated phytoremediation capabilities, particularly in the uptake of zinc, copper, and manganese from leachate [29]. This grass species is known for its good biomass yield even under marginal soil and water conditions and exhibits tolerance to salinity [19]. Comparative studies have also shown that Rhode grass accumulates higher concentrations of essential micronutrient metals, such as copper, iron, manganese, and zinc, compared to oat [19].

*Setaria sphacelata* has also appeared to be particularly effective in the phytoextraction of multiple micronutrients from contaminated soils in Ethiopia [19]. The findings of studies suggest that it surpasses oat in the accumulation of copper, iron, manganese, and zinc and is the most efficient in the accumulation of micronutrients per unit of soil. Continually, *Arundo donax* (giant reed) has shown a specific effectiveness in the phytoremediation of Cr (VI) from soil contaminated by tannery wastewater, with a TF greater than 1 mg kg^−1^ indicating its potential for phytoextraction [20]. Additionally, this species has demonstrated the ability to absorb Cd, Cr, and Pb from the Little Akaki River in Ethiopia [21].

Beyond these extensively studied grasses, the study also found other Ethiopian plants, including several grass species, that exhibited phytoremediation potential. These include *Argemone mexicana*, *Rumex nepalensis*, *Cyperus alopecuroides*, and *Schoenoplectus confusus*. The results have also shown promise for the phytoextraction of copper and nickel in the vicinity of the Legadembi tailings dam [16]. *Rumex nepalensis*, *Cyperus alopecuroides*, and *Typha latifolia* also possess the ability to phytostabilize zinc in the same area [16]. *Saccharum bengalense* has shown potential for phytostabilization of red mud deposits [30]. Guinea grass (*Panicum maximum*) appears promising for the phytoremediation of soils contaminated with Pb, Cr, and Cd [22]. Desho grass (*Pennisetum pedicellatum*) and elephant grass (*Pennisetum purpureum*) are effective in stabilizing the soil bund and erosion control, and elephant grass has also shown copper uptake from leachate [29]. Chomo grass (*Brachiaria humidicola*) plays a role in the rehabilitation of degraded land and in the improvement of soil fertility [25], while *Setaria incrassata* is used for erosion control [19]. Furthermore, Guassa grasses (*Festuca* spp.) are important for local community uses, and specific *Festuca* species such as *Festuca rubra* and *Festuca pratensis* have shown potential for zinc phytostabilization [31].

The reviewed studies have indicated that various native grass species in Ethiopia employ different phytoremediation mechanisms. Phytoextraction, the accumulation of pollutants in the aboveground biomass, harvestable parts of the plant, and phytostabilization, the immobilization of pollutants in the soil, often within the root zone, are the primary mechanisms observed [18]. Some species, such as Vetiver grass, exhibit the potential for both mechanisms depending on the specific pollutant and environmental conditions. Furthermore, the role of phytoliths, silica bodies produced by some plants, has been noted in the immobilization of Pb in contaminated soils [32].

The effectiveness of these grass species is often quantified using BCF and TF values. A BCF greater than 1 mg kg^−1^ typically indicates that the plant has the ability to accumulate the pollutant in its tissues, while a TF greater than 1 mg kg^−1^ suggests that the pollutant is efficiently transported from the roots to the shoots, which is particularly desirable for phytoextraction [16]. In contrast, a BCF greater than 1 but a TF less than 1 mg kg^−1^ often indicates that the plant is more suitable for phytostabilization, as it retains the pollutant primarily in its roots.

#### 3.1.1. Factors Affecting the Effectiveness of Grasses in Phytoremediation

The findings of the articles reviewed aligned with the broader global understanding of phytoremediation, which recognizes the significant potential of grasses for the treatment of sites contaminated with heavy metals and other pollutants [15]. Specific examples and data emerging from studies conducted in Ethiopia contribute valuable localized insights to this global knowledge base. The identification of various native grass species with demonstrated phytoremediation capabilities offers promising, potentially sustainable, and cost‐effective solutions for addressing the diverse contamination issues prevalent in different regions of Ethiopia, including areas affected by mining, industrial activities, and agricultural runoff. The utilization of these native species holds an advantage over the introduction of nonnative plants, as they are already adapted to local environmental conditions and can contribute to the restoration of native ecosystems.

However, the success of phytoremediation using native grasses in Ethiopia is influenced by a complex interplay of factors. These include the specific characteristics of the grass species itself, such as its growth rate, biomass yield, root system architecture, and tolerance to the target pollutant [15]. The type and concentration of the pollutant are also critical determinants of the efficacy of phytoremediation, as different plants exhibit varying affinities and tolerance levels for different contaminants [33]. Soil characteristics, such as pH and organic matter content, play a significant role in the bioavailability of pollutants and consequently affect their uptake by plants [19]. Climatic conditions, including rainfall and temperature, can also influence plant growth and thus the overall success of phytoremediation efforts. Furthermore, the chosen phytoremediation mechanisms, whether phytoextraction or phytostabilization, dictate the management strategies employed and the ultimate outcome of the remediation process [15].

Although phytoremediation offers numerous advantages, including its cost‐effectiveness, environmental friendliness, and applicability as an in situ treatment [15, 34], it is not without limitations. The process can be time‐consuming, often requiring multiple growing seasons to achieve a significant reduction in contaminant [20]. The depth to which plant roots can penetrate may limit the treatment zone, particularly for pollutants located deeper in the soil profile [29]. Furthermore, if not managed properly, pollutants accumulated in plant tissues are likely to enter the food chain, posing risks to herbivores and ultimately humans [14, 15]. Therefore, careful selection of plant species and appropriate management practices, such as harvesting and disposal of contaminated biomass, are crucial to ensuring the safe and effective application of phytoremediation in Ethiopia.

### 3.2. Environmental Pollution in Ethiopia

Environmental pollution manifests itself today in Ethiopia in several aspects as it continues to develop into an industrial and agricultural nation. This form of pollution arises not only from mining activities through tailing dams and draining of potentially toxic elements, such as Pb, Ni, Cu, Zn, and Cd, into adjacent soils and water bodies [10, 11] but also comes from industrial and urbanized areas like Addis Ababa and nearby areas such as the Akaki River Basin, where untreated waste effluents endowed with heavy metals (Cr, Pb, Cd, Ni, Cu, Zn, etc.) and other pollutants are discharged from tanneries, textile factories, and metal workshops [35]. Much of this agricultural activity contributes further through the application of pesticides and fertilizers, while various specific processes, such as wet coffee processing, generate wastewater for treatment [18].

Several areas in Ethiopia are severely salt‐affected, such as the Rift Valley and lowlands, such as Raya Kobo, where some soil salinity and sodicity are a regular feature, in many cases aggravated with irrigation from marginal quality water and inadequate drainage practice [36]. Such salt‐affected soils cover millions of acres and threaten agricultural productivity and livelihoods [37].

### 3.3. Phytoremediation Practices in Ethiopia

The high rates of contamination challenges in Ethiopia are derived from sources at points. Heavy metals such as Cr, Pb, Cd, and As are mainly found close to industrial zones within tanneries and mining areas and originate from unregulated waste disposal [38, 39]. Farmers also contribute by applying fertilizer and pesticides, where some residues of persistent organic pesticides such as DDT have remained in some areas even now [28].

Phytoremediation is a collection of methods through which plants remediate contaminants. For example, phytoextraction is the uptake of contaminants by the roots into the plant from soil/water, followed by the consequent translocation to the harvestable shoots [40]. On the other hand, phytostabilization is the absorption of contaminants by the roots and adsorption to the surface of the roots. It also involves the production of biochemicals by plants, which are then released into the soil or groundwater around the roots. Biochemicals have the ability to sequester, precipitate, or otherwise immobilize nearby contaminants [41]. Rhizofiltration is the term that refers to the adsorption or precipitation of contaminants in plant roots and is mainly used for water treatment [42]. The efficiency of this process is best determined by the coefficients of [TF] = [shoot concentration] / [root concentration], and [BCF] = [root concentration] / [soil concentration. Generally, TF > 1 and BCF (or biological accumulation coefficient [BAC]) > 1 imply suitability for phytoextraction, while BCF > 1 and TF < 1 hold for phytostabilization [18, 40]. Phytodegradation refers to the absorption of very complex organic pollutants into less complex and less toxic molecules by metabolic processes within plant tissues or by the action of enzymes exuded by plant roots [43]. And rhizodegradation is nearly similar, except that it considers enhancing the microbial degradation of organic pollutants in the rhizosphere—the soil zone directly influenced by plant roots—because of the exudation of nutrients and enzymes by the plant [44]. Finally, phytovolatilization is the uptake of some contaminants by plants (mercury, selenium, and some organic solvents), changing the form in which they are released into the atmosphere to a more volatile and often less harmful form [45].

### 3.4. Grass Species Used for Phytoremediation in Ethiopia

Ethiopia has remarkable biodiversity with a huge variety of species of grasses that fit into its various agroecological zones, from the very dry lowlands to the cool highlands [46]. These native grass species represent a very useful yet underexplored area for phytoremediation research. Grasses exhibit certain characteristics that adequately support their candidacy for phytoremediation. Their typically dense and fibrous root systems act efficiently to anchor soil, thus preventing soil erosion while presenting a very high surface area for interaction with soil contaminants, facilitating both uptake (phytoextraction) and immobilization (phytostabilization) [47]. Furthermore, many grass species grow and develop rapidly while producing huge biomass, facilitating phytoextraction efficiency (which requires mass removal of contaminants) and the establishment of quick ground cover in phytostabilization scenarios [48]. Native grasses may have some level of tolerance to abiotic stress, including drought, poor soil fertility, and possibly moderate levels of contamination; conditions are often met in polluted sites [49, 50]. Although the selection is not performed thoroughly, promising Ethiopian native genera would include, based on the distribution, hardiness, and known attributes of related species around the world, *Hyparrhenia*, *Pennisetum* (species that are currently commonly classified under *Cenchrus*), *Cynodon*, *Eragrostis*, *Chloris*, and *Sporobolus*. Their integrated ecological adaptation suggests that native species will outperform those introduced that are lower in input and higher in ecological compatibility.

#### 3.4.1. Biological Characteristics of Grasses Relevant to Phytoremediation

Few native grasses show essential features for phytoremediation. Different grasses have a fibrous root system that spreads extensively to explore volumes of soil to enhance contact with contaminants while providing considerable surface area for adsorption and uptake [51]. Some species, such as *Vetiveria zizanioides*, are characterized by exceptionally long roots that reach an average depth of about 3 m, which makes the grass useful for stabilization, erosion control, and even accessing deeper rooted contaminants [10]. Furthermore, grasses are known to grow rapidly and produce biomass (e.g., Napier grass, *Cenchrus purpureus*), which promotes the phytoextraction of pollutants stored in aboveground biomass, which can be further harvested [51].

Vetiver grass is tolerant to drought, salinity, and a wide range of heavy metals and pH conditions [10, 18]. These halophytic grasses, such as Rhodes grass (*Chloris gayana*) and Bermuda grass (*Cynodon dactylon*), are specialized for saline conditions and thus can be used to remedy salt‐affected soils [36]. Other native species include several *Cyperus* (grasses, usually grass‐like) and *Typha latifolia* (cattail), *Rumex nepalensis*, *Phytolacca dodecandra*, *Adhatoda schimperiana*, and *Solanum incanum*, all of which have unique characteristics that would make them suitable for the uptake or stabilization of specified contaminants [11]. Many grasses plus a few shrub species are perennials, making them a potential avenue for long‐term remediation through regeneration and repeated harvesting [40].

The suitability of plants for phytoremediation depends on a combination of physiological and morphological characteristics. Native Ethiopian grasses and other plants studied exhibit several characteristics, as listed below, which make them strong candidates.

#### 3.4.2. Nature of the Root System

**Figure 2:**
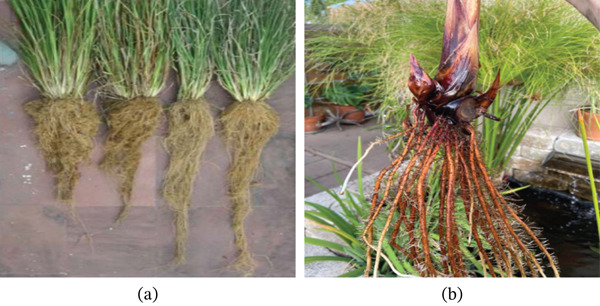
(a, b) Nature of the root system of grasses. (a) *Chrysopogon zizanioides*. (b) *Cyperus papyrus*.

Grasses are defined by their fibrous root systems (Figure 2), which create a dense network in the upper soil layers, maximizing soil exploration and contact with contaminants [51]. Vetiver grass (*Vetiveria zizanioides*) is exceptional, having a massive and finely structured root system that can penetrate several meters deep, providing unparalleled soil stabilization and access to deeper pollutants [10, 18]. Species such as *Typha latifolia* and *Cyperus papyrus* also have well‐developed rhizomes and root systems adapted to wetland conditions.

#### 3.4.3. Biomass Production and Growth Rate

**Figure 3:**
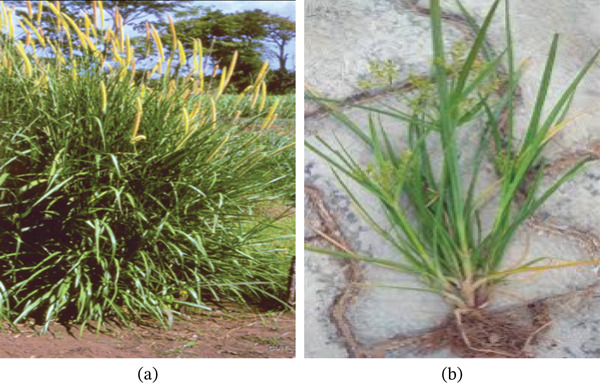
(a, b) Biomass production and growth rate of grass. (a) *Cenchrus purpureus*. (b) *Cyperus alopecuroides*.

Rapid growth and high accumulation of biomass are crucial for effective phytoextraction. Napier grass (*Cenchrus purpureus*) is renowned for its high productivity [51]. Vetiver grass also produces significant biomass [18]. Napier grass has the highest Zn uptake and good Cr accumulating capacity [52]. The Cr uptake rate increased in Napier grass with increasing plant age [52]. This allows greater contaminant removal per harvest cycle (Figure 3). Many of these are perennials that regrow after cutting, enabling long‐term management [22].

#### 3.4.4. Stress‐Tolerant Grass Species

**Figure 4:**
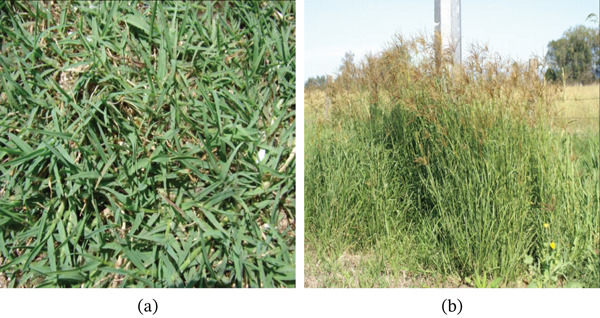
(a, b) Stress‐tolerant grass species. (a) *Cynodon dactylon*. (b) *Chloris gayana*.

Native plants are often adapted to local environmental stresses. Vetiver grass exhibits remarkable tolerance to drought, salinity, extreme pH, and high concentrations of various heavy metals [10]. Halophytic grasses, such as *Chloris gayana* (Rhodes grass) and *Cynodon dactylon* (Bermuda grass), are specifically adapted to thrive in saline‐sodic soils [36]. Studies on *Phytolacca dodecandra*, *Adhatoda schimperiana*, and *Solanum incanum* showed that they could grow vigorously at highly contaminated industrial sites in Ethiopia [40]. Tolerance allows plants to survive and function metabolically, even when they accumulate potentially toxic substances (Figure 4).

#### 3.4.5. Accumulation and Translocation Ability

The core of phytoremediation lies in the ability to uptake, translocate, accumulate, and degrade contaminants. Different species show varying affinities for different metals. Key indicators are the BCF (root uptake from soil), the TF (root‐to‐shoot movement), and the BAC (shoot uptake from soil) [18]. Values > 1 mg kg^−1^ for these factors indicate the potential for accumulation and translocation relevant to phytoextraction and phytostabilization [40]. Specific examples studied in Ethiopia include *Rumex nepalensis*, *Cyperus alopecuroides*, *Typha latifolia*, *Schoenoplectus confusus*, *Polygonum coccineum*, *Brachiaria mutica*, *Cyperus papyrus*, *Chrysopogon zizanioides*, *Chloris gayana*, *Cynodon dactylon*, *Setaria sphacelata*, *Phytolacca dodecandra*, *Adhatoda schimperiana*, *Solanum incanum*, and *Argemone mexicana* [36].

## 4. Future Perspectives and Research Gaps

Several studies highlight the need for in situ field–based research in Ethiopia to authenticate results from laboratory and pot experiments [20]. Given the country′s diverse agroecological conditions, field trials are essential to assess the effectiveness and adaptability of native grass species. There is also a need to expand research within the sub‐Saharan African context [18]. Although some native grasses have shown potential, Ethiopia′s biodiversity is expected to include additional species with unused phytoremediation potential. Future studies should therefore focus on systematic species identification and functional evaluation of valuable species for their ability to remediate a wider range of pollutants.

To improve the efficiency and applicability of phytoremediation in Ethiopia, future studies could focus on improving existing techniques. This includes exploring the use of various soil amendments to increase pollutant bioavailability and plant uptake, investigating the potential of microbial assistance and the use of mycorrhizal fungi or pollutant‐degrading bacteria, and improving phytoremediation processes in pollutant accumulation or degradation capabilities [15]. Assessing the long‐term sustainability and broader ecological impacts of phytoremediation efforts using native grasses in Ethiopia is another critical area for future research. Long‐term studies are needed to assess the sustainability and ecological impacts, including effects on soil health, biodiversity, and ecosystem functioning [23].

Finally, the successful implementation of phytoremediation strategies in Ethiopia requires thorough consideration of socioeconomic factors. In addition, socioeconomic factors such as farmer adoption, land use, and cost–benefit analysis should be integrated to support practical implementation [25]. Specific recommendations for future studies include standardized measurement and reporting of BCF and TF, use of controlled and field‐based experimental designs, inclusion of appropriate controls and replicates, soil and plant tissue analysis protocols, and integration of physicochemical parameters. Understanding the perceptions and needs of local communities is essential for the widespread and effective adoption of these sustainable remediation practices.

## 5. Conclusions

This systematic review synthesized available evidence on the phytoremediation potential of grass species in Ethiopia, based on literature retrieved from PubMed, Scopus, Web of Science, and Google Scholar. A total of 17 indigenous grass and plant species have been identified for their potential use. The results found that *Chrysopogon zizanioides* (Vetiver grass), *Rumex nepalensis*, *Chloris gayana*, *Cyperus* species, *Cynodon dactylon*, *Typha latifolia*, and *Brachiaria mutica* are highly used for phytoremediation. This grass species demonstrated capabilities for phytoextracting and phytostabilizing specific contaminants. The choice of species and the dominant mechanisms depend heavily on the target contaminant and local environmental factors. Grasses, with their vigorous root systems and stress tolerance, appear to be particularly well suited for phytostabilization and soil improvement. Certain species showed high TFs (TF > 1 mg kg^−1^) for phytoextraction of specific metals such as Cu and Ni. Ethiopia can efficiently use its native botanical resources to develop sustainable, cost‐effective, and ecologically sound solutions to environmental remediation challenges.

Overall, grasses with extensive root systems and high stress tolerance appear particularly suitable for phytostabilization and soil rehabilitation, while some species show potential for phytoextraction, as indicated by relatively high TFs for metals such as Cu and Ni. However, the current evidence is largely derived from controlled or limited studies, highlighting significant gaps in field validation, long‐term performance assessment, and ecological impact evaluation. Therefore, while grass species show promising potential, their large‐scale application in Ethiopia remains constrained by insufficient in situ research and methodological standardization. Future studies should focus on field‐based validation, optimization of phytoremediation techniques, and integration of ecological and socioeconomic considerations to ensure practical and sustainable implementation.

## Author Contributions

Endalamaw Yihune conceived the study, conducted the primary literature search, synthesized and analyzed the data, and wrote the initial draft of the manuscript. As the corresponding author, Endalamaw Yihune handled all communications and revisions. Amare Bitew Mekonnen contributed to the editing of the manuscript. Getahun Yemata provided expert input on the phytoremediation uses of native grasses in Ethiopia and contributed to the review and editing of the manuscript.

## Funding

No funding was received for this manuscript.

## Conflicts of Interest

The authors declare no conflicts of interest.

## Data Availability

The authors are volunteers who provide all data used upon a justifiable request to the corresponding author.
